# Evaluation of Endocrine Late Complications in Childhood Acute Lymphoblastic Leukemia Survivors: A Report of a Single-Center Experience and Review of the Literature

**DOI:** 10.4274/tjh.2015.0332

**Published:** 2017-03-01

**Authors:** Cengiz Bayram, Neşe Yaralı, Ali Fettah, Fatma Demirel, Betül Tavil, Abdurrahman Kara, Bahattin Tunç

**Affiliations:** 1 Ankara Children’s Hematology and Oncology Hospital, Clinic of Pediatric Hematology, Ankara, Turkey; 2 Private Doctor

**Keywords:** Acute lymphoblastic leukemia, Endocrine, Late effects, children

## Abstract

**Objective:**

Improvement in long-term survival in patients with acute lymphoblastic leukemia (ALL) in childhood has led to the need for monitorization of treatment-related morbidity and mortality. In the current study, we aimed to evaluate endocrine side effects of treatment in ALL survivors who were in remission for at least 2 years.

**Materials and Methods:**

Sixty patients diagnosed with ALL, who were in remission for at least 2 years, were cross-sectionally evaluated for long-term endocrine complications.

**Results:**

The median age of the patients at the time of diagnosis, at the time of chemotherapy completion, and at the time of the study was 5 years (minimum-maximum: 1.7-13), 8 years (minimum-maximum: 4.25-16), and 11.7 years (minimum-maximum: 7-22), respectively, and median follow-up time was 4 years (minimum-maximum: 2-10.1). At least one complication was observed in 81.6% of patients. Vitamin D insufficiency/deficiency (46.6%), overweight/obesity (33.3%), and dyslipidemia (23.3%) were the three most frequent endocrine complications. Other complications seen in our patients were hyperparathyroidism secondary to vitamin D deficiency (15%), insulin resistance (11.7%), hypertension (8.3%), short stature (6.7%), thyroid function abnormality (5%), precocious puberty (3.3%), and decreased bone mineral density (1.7%). There were no statistically significant correlations between endocrine complications and age, sex, and radiotherapy, except vitamin D insufficiency/deficiency, which was significantly more frequent in pubertal ALL survivors compared to prepubertal ALL survivors (57.5% and 25%, respectively, p=0.011).

**Conclusion:**

A high frequency of endocrine complications was observed in the current study. The high frequency of late effects necessitates long-term surveillance of this population to better understand the incidence of late-occurring events and the defining of high-risk features that can facilitate developing intervention strategies for early detection and prevention.

## Abstract (tr)

**Amaç:**

Akut lenfoblastik lösemili (ALL) hastalardaki sağ kalım oranlarının artışı, tedavi sonrası ortaya çıkan morbidite ve mortalite problemlerinin takip edilme ihtiyacını beraberinde getirmiştir. Çalışmamızda, en az iki yıldır remisyonda olan ALL’li hastalarda, tedavi sonrası ortaya çıkabilecek endokrin komplikasyonların değerlendirilmesi amaçlandı.

**Gereç ve Yöntemler:**

ALL tanısı ile tedavi almış ve tedavisi üzerinden en az iki yıl geçmiş ve remisyonda olan 60 hastada endokrin geç komplikasyonlar kesitsel olarak değerlendirildi.

**Bulgular:**

Hastaların tanı aldıkları andaki median yaşları 5 yıl (minimum-maksimum: 1,7-13), kemoterapi sonlandırıldığı andaki median yaşları 8 yıl (minimum-maksimum: 4,25-16), çalışma sırasındaki median yaşları ise 11,7 yıl (minimum-maksimum: 7-22) olarak tespit edildi. Hastaların tedavi sonrası median takip süresi ise 4 yıl (minimum-maksimum: 2-10,1) idi. Hastaların %81,6’sında en az bir endokrin komplikasyon geliştiği görüldü. D vitamini eksikliği/yetersizliği (%46,6), obezite/fazla kiloluluk (%33,3) ve dislipidemi (%23,3) en sık gelişen üç komplikasyon olarak tespit edildi. D vitamini eksikliğine sekonder gelişen hiperparatiroidi (%15), insülin direnci (%11,7), hipertansiyon (%8,3), boy kısalığı (%6,7), tiroid fonksiyon bozukluğu (%5), puberte prekoks (%3,3) ve azalmış kemik mineral yoğunluğu (%1,7) gelişen diğer endokrin komplikasyonlardı. Hastalarda gelişen endokrin komplikasyonlar arasında cinsiyet, yaş, radyoterapi bakımından farklılık saptanmaz iken, D vitamini yetersizliği/eksikliği saptanan hasta sayısı pubertal grupta, prepubertal gruba göre anlamlı derecede fazlaydı (%57,5 ve %25, sırasıyla, p=0,011).

**Sonuç:**

Çalışmamızda yüksek oranda endokrin komplikasyon saptandı. Bu komplikasyonlar, geç yan etkilerin ortaya çıkmasına neden olabilecek yüksek risk özellikleri ve sıklığı tanımlayabilmek, erken tanı ve önleyici stratejileri geliştirmek açısından hastaların uzun dönem izlenmelerini gerektirmektedir.

## INTRODUCTION

Despite the increase in the prevalence of childhood malignancies, the 5-year survival rate for acute lymphoblastic leukemia (ALL) in childhood has approached 90% as a result of advances in chemotherapy and supportive care. This increase in survival has increased the importance of long-term treatment-related morbidity and mortality [1]. One of the consequences of cancer or its therapy is that many long-term survivors of childhood cancer are at an increased risk of developing chronic physical or psychosocial problem [2]. It is estimated that about two-thirds of cancer survivors will experience at least one late adverse effect and more than 40% may have a severe, disabling, or life-threatening condition or may die 30 years after the cancer is diagnosed [3]. There have been numerous chemotherapy agents used for the treatment of ALL; however, a few of them have been implicated as causing late effects, including anthracyclines (e.g., doxorubicin, daunorubicin), oxazaphosphorine alkylating agents (e.g., cyclophosphamide), corticosteroids (e.g., prednisone, dexamethasone), and high-dose methotrexate [2]. The adverse effects of prophylactic cranial irradiation, including acute neurotoxicity, neurocognitive deficits, endocrinopathies, secondary malignant disease, and excess late mortality, have led to its reduction or elimination from treatment protocols for ALL [4,5,6].

Endocrine complications during therapy for ALL include bone demineralization, disordered growth, adrenocortical insufficiency, diabetes mellitus, the syndrome of inappropriate secretion of antidiuretic hormone, and changes in thyroid hormone concentration, whereas late complications, those that occur after completion of all radiation and chemotherapy, include bone demineralization, short stature, growth hormone deficiency, obesity, hypothyroidism, gonadal dysfunction, and infertility [7]. The present study aimed to evaluate long-term endocrine complications in ALL survivors that were in remission for at least 2 years.

## MATERIALS AND METHODS

Sixty patients diagnosed with ALL between January 2003 and February 2009 at the Pediatric Hematology Clinic of Ankara Children’s Hematology and Oncology Education and Research Hospital, who were in remission for at least 2 years, were included in the study and were evaluated cross-sectionally. All patients with ALL were treated according to the St. Jude Total-XIIIA protocol [8] and received 12 or 18 cGy cranial radiotherapy (CRT) as a part of prophylaxis or treatment as appropriate.

Pubertal status was assessed at the time of the study using Tanner staging and patients were divided into two groups as pubertal or prepubertal. Body weight and height were measured and evaluated according to Turkish children’s growth data [9]. Body mass index (BMI) was calculated as weight in kilograms divided by the square of the height in meters. Overweight was defined as BMI for age and sex between the 85^th^ and 95^th^ percentiles, and BMI for age and sex higher than the 95^th^ percentile was defined as obesity.

Hormonal analysis was carried out by the chemiluminescence method using a BIO-DPC hormone autoanalyzer with commercial kits, and a Roche/Hitachi Modular P Chemistry Analyzer was used for biochemical analysis. Serum lipid profiles, including cholesterol, triglyceride, low-density lipoprotein cholesterol (LDL-C), high-density lipoprotein cholesterol (HDL-C), and blood glucose, were obtained after at least 8 h of fasting. Dyslipidemia was defined as cholesterol of >200 mg/dL, or triglyceride of >150 mg/dL, or LDL-C of >130 mg/dL, or HDL-C of <40 [10]. The homeostasis model assessment (HOMA) score was calculated using the following formula: [fasting glucose (mmol/L) × insulin (mmol/L)]/22.5. A HOMA score above 2.5 for prepubertal patients and above 3.2 for pubertal patients was accepted as evidence of insulin resistance [11,12]. Metabolic syndrome was defined as the presence of three or more of the following: 1) hypertension, 2) glucose intolerance, 3) hypertriglyceridemia, 4) decreased HDL level, and 5) central abdominal obesity [13]. Low free thyroxine (T4) and thyroid-stimulating hormone (TSH) level of >10 µU/mL with clinical symptoms were defined as hypothyroidism, and normal free T4 level and TSH level between 5 and 10 µU/mL without clinical symptoms were defined as subclinical (compensated) hypothyroidism [14]. Parathyroid hormone (PTH) values between 6 and 65 pg/mL were accepted as normal. High PTH levels with vitamin D deficiency were diagnosed as “secondary hyperparathyroidism”. Patients having hypocalcemia, hyperphosphatemia, and decreased PTH levels were diagnosed with “primary hypoparathyroidism”. Serum adrenocorticotropic hormone (ACTH) and cortisol levels were simultaneously measured at 09:00 hours. ACTH levels between 7 and 28 pg/mL for prepubertal children and between 2 and 49 pg/mL for postpubertal children were accepted as normal. Cortisol levels between 8 and 22 µg/dL were accepted as normal, whereas <8 µg/dL was accepted as adrenal insufficiency [15]. Serum 25-OH-vitamin D levels less than 15 ng/mL were diagnosed as a sign of vitamin D deficiency (normal: 20-100 ng/mL) and values between 15 and 20 ng/mL were considered as vitamin D insufficiency [16]. Bone mineral density (BMD) was measured by using dual energy X-ray absorptiometry from the L1-L4 lumbar vertebrae and was assessed according to bone ages appropriately for Turkish children’s data [17]. Age- and sex-adjusted Z-scores of less than -2 were considered as evidence of decreased BMD.

The study was approved by the hospital ethics committee, and informed consent was obtained from the parents of all participating subjects.

Continuous variables are expressed as median (minimum-maximum) and categorical variables as number (percentage). The clinical parameters and laboratory values of the patients were evaluated using the chi-square method and Student’s t-test, as appropriate. All statistical tests were two-sided and p<0.05 was considered statistically significant. Statistical analysis was performed using SPSS 18.0 for Windows (SPSS Inc., Chicago, IL, USA).

## RESULTS

Of the 60 ALL survivors, 31 (51.7%) were male and 29 (48.3%) were female. Fifty-five patients (91.7%) were diagnosed with pre-B-cell ALL and five (8.3%) were diagnosed with T-cell ALL. Overall, 21 patients (35%) received 12 or 18 cGy CRT as a part of prophylaxis or treatment as appropriate. The median age of the patients at the time of diagnosis, at the time of chemotherapy completion, and at the time of the study was 5 years (minimum-maximum: 1.7-13), 8 years (minimum-maximum: 4.25-16), and 11.7 years (minimum-maximum: 7-22), respectively, and median follow-up time was 4 years (minimum-maximum: 2-10.1). Demographic data of patients are summarized in Table 1.

One or more adverse events occurred in 81.6% of the 60 ALL survivors; 25 patients (41.6%) had one endocrine complication, 17 patients (28.3%) had two endocrine complications, 5 patients (8.3%) had three endocrine complications, and 2 patients (3.3%) had four endocrine complications. Vitamin D insufficiency/deficiency (46.6%), overweight/obesity (33.3%), and dyslipidemia (23.3%) were the three most frequent endocrine complications, followed by hyperparathyroidism secondary to vitamin D deficiency (15%), insulin resistance (11.7%), hypertension (8.3%), short stature (6.7%), thyroid function abnormality (5%), precocious puberty (3.3%), and decreased BMD (1.7%). There were no patients diagnosed with adrenal insufficiency, as serum ACTH and cortisol levels were within the normal ranges for all patients (Table 2).

There were four patients (6.6%) with three components of metabolic syndrome, seven patients (11.6%) with two components of metabolic syndrome, and 18 patients (30%) with one component of metabolic syndrome. Of the seven patients with insulin resistance, five had overweight or obesity, and two patients had normal BMI.

In the present study, long-term endocrine complications in 60 ALL survivors were also assessed with respect to age (<5 years versus >5 years), sex (male versus female), pubertal status (pubertal versus prepubertal), and either CRT administered or not. No significant differences were observed with respect to age, sex, and cranial radiotherapy, whereas vitamin D insufficiency/deficiency was significantly more frequent in pubertal ALL survivors (57.5%) as compared to prepubertal ALL survivors (25%) (Table 3).

## DISCUSSION

Despite the increase in the prevalence of childhood cancer, with a 0.6% increase in incidence rates for all childhood cancers noted during 1975-2002, a reduction in the mortality rate has been achieved through multimodal chemotherapy and enhanced supportive care [18]. However, as a consequence of treatment-related complications, recurrence of primary cancer, and subsequent malignancies, approximately two-thirds of this population is reported to have one or more late adverse effects [18,19,20,21]. In concordance with previous reports, one or more adverse events occurred in 81.6% of the present study’s ALL survivors. Vitamin D insufficiency/deficiency (46.6%) was the most frequent endocrine complication in the current study. ALL survivors are at increased risk of developing BMD deficits compared to the general population, associated with their treatment, including high cumulative doses of steroids/methotrexate and radiation therapy. Because vitamin D has a positive influence on calcium balance for building bone and attaining peak bone mass, vitamin D deficiency or insufficiency can contribute to BMD deficit [22]. The prevalence of 25-hydroxyvitamin D (25-OH-D) insufficiency is reported to be 14-49% in the general population and was reported to be between 33.5% and 40% in healthy Turkish children and adolescents in two recent studies [23,24,25,26]. In a recent study of 484 childhood cancer survivors, 17.6% of whom had leukemia, Choudhary et al. reported a prevalence of 29% of 25-OH-D insufficiency, and the risk factors for 25-OH-D insufficiency were race, pubertal status, and seasonality [27]. The prevalence of 25-OH-D deficiency or insufficiency was 46.6% in our study, which was higher than in the latter study and two recent studies from Turkey but similar to what has been described in the general population. Pubertal status was the only significant risk factor in the present study, while 23 of 28 patients (82.1%) with 25-OH-D deficiency or insufficiency were pubertal ALL survivors. Forty percent of bone mass is obtained during puberty, and by the end of puberty, 90% of total adult bone mass has already been acquired in the normal population [22]. In this regard, in addition to chemotherapy agents and radiation therapy, 25-OH-D deficiency or insufficiency can additionally contribute to failure to recover to a normal BMD after completion of therapy, and thus surveillance and intervention strategies should also include assessment of 25-OH-D levels during puberty.

Overweight or obesity has been identified as a potential late adverse effect of therapy in childhood ALL survivors [2,3]. The largest study evaluating the risk of being overweight in ALL survivors was conducted by the Childhood Survivor Cancer Study. That study showed that survivors who received greater than 20 Gy CRT were significantly more likely than their siblings to be overweight, and female survivors treated before the age of 4 years were also more likely to be overweight in comparison with siblings [28]. Studies about the risk of being overweight or obese in ALL survivors have conflicting results. In a study of 618 ALL survivors, reported by Dalton et al., they found that children treated before the age of 13 years had a significant decrease in their height z-scores and an increase in their BMI z-scores, regardless of cranial radiation therapy [29]. Razzouk et al. observed that young age (<6 years) and overweight/obesity at diagnosis were the best predictors of obesity at adult height in a study of 456 childhood ALL and lymphoma survivors, 431 of whom had ALL [30]. That study further concluded that BMI weight category at diagnosis, rather than type of CNS treatment received, predicted adult weight in long-term survivors of childhood hematologic malignancies. Zhang et al. reported that weight status at diagnosis and BMI z-score at diagnosis were both significant predictors for being overweight/obese at the end of treatment in pediatric ALL survivors [31]. They found that patients who were overweight/obese at diagnosis were 11.9 times more likely to be overweight/obese at the end of treatment than those who were underweight or had healthy weight at diagnosis. Moreover, sex, receiving CRT, and age at diagnosis were not significant predictors of BMI z-score in survivors of pediatric ALL during or after treatment. In the present study, we also did not observe any significant correlation between the risk of being overweight or obese and age at diagnosis, CRT, and sex. Additionally, Asner et al. showed an association between abnormal maternal BMI and overweight/obesity in long-term survivors of childhood ALL, except for overweight/obesity at diagnosis, while age at diagnosis, sex, cumulative dose of steroids, and paternal BMI showed no association [32]. In a previous study of 33 ALL survivors, 25 of whom were female, it was found that 56% of female survivors were obese and 59% of them had an obese mother [33]. Considering the risk of long-term excessive weight gain in childhood ALL survivors including overweight/obesity at diagnosis and abnormal maternal BMI, rather than CRT, corticosteroids, age at diagnosis, and sex, the patient’s familial and genetic characteristics are likely to be risk factors leading to overweight/obesity for this population.

ALL survivors are reported to have a 4-fold excess risk of mortality secondary to cardiovascular events compared to the general population. In addition to cardiomyopathy associated with anthracyclines, ALL survivors have also been shown to have atherosclerotic disease, which led to an investigation of conventional risk factors for cardiovascular disease including diabetes mellitus, dyslipidemia, obesity, and metabolic syndrome [13]. In the present study, the second and third most common endocrine complications were overweight/obesity (33.3%) and dyslipidemia (23.3%), whereas insulin resistance (11.7%) and hypertension (8.3%) were the fourth and fifth most common endocrine complications. Because childhood ALL survivors will be young at the time of completion of treatment, as in the present study (median age: 11.7 years), many treatment-related adverse events may not become clinically apparent until the survivor gets older in the context of cardiovascular disease development, and thus preventive strategies including medical interventions and lifestyle modifications such as eating a healthful diet, regular physical activity, and avoiding cancer-associated habits including smoking or excessive alcohol consumption can help reduce the risk factors leading to cardiovascular late events.

ALL survivors treated with conventional CRT doses do not usually develop other central endocrinopathies, such as central adrenal insufficiency, hyperprolactinemia, gonadotropin insufficiency, or central (secondary) hypothyroidism. However, primary hypothyroidism can occur after cranial, craniospinal, and total body irradiation because of direct exposure of the thyroid gland to radiation, even at low doses [2]. Precocious puberty is another late effect of CRT in doses of 18 to 24 Gy, and it is more common in girls. However, most female ALL survivors experience menarche at a normal age, which was confirmed in two large cohorts [34,35]. Reduction or elimination of CRT in treatment of childhood ALL in recent protocols is another reason for favorable outcome in the context of central endocrinopathy development [4,5,6]. In the present study, there were no patients diagnosed with adrenal insufficiency, and the frequency of patients with hypothyroidism and subclinical hypothyroidism (compensated) was 1.7% and 3.3%, respectively. Only two of 60 ALL survivors developed precocious puberty; both were girls and one received CRT.

## CONCLUSION

In conclusion, a high frequency of endocrine long-term adverse events occurred in the current study. In achieving >90% of 5-year survival rates for children with ALL diagnosed at 14 years of age or younger, there has been an increase in the number of children and adolescents cured of ALL. In this context, considering the high prevalence of late adverse effects as a consequence of ALL or its therapy as compared to the general population, long-term surveillance of this population is important to better understand the incidence of late-occurring events and define high-risk features that can facilitate the development of intervention strategies for early detection and prevention, which can lead to an improvement of care and quality of life for this growing population.

## Figures and Tables

**Table 1:**
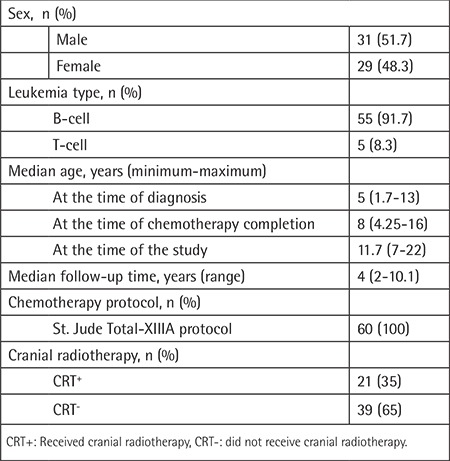
Patient characteristics.

**Table 2:**
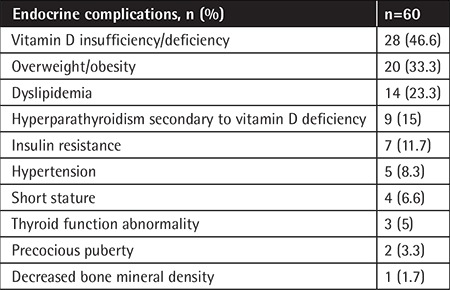
Endocrine complications.

**Table 3:**
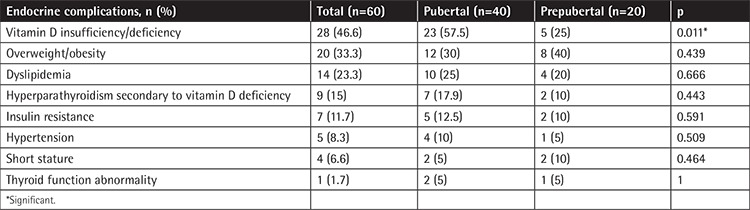
Correlation between endocrine complications and pubertal status.
